# A Rare Case of Multiple Bone Infarctions and Abnormal Pulmonary Function Tests in a Patient With Compound Heterozygous Hemoglobin S and Type 2 Hereditary Persistence of Fetal Hemoglobin

**DOI:** 10.7759/cureus.66395

**Published:** 2024-08-07

**Authors:** Khalid A Alnaqbi

**Affiliations:** 1 Internal Medicine, College of Medicine and Health Sciences, United Arab Emirates University, Al Ain, ARE; 2 Rheumatology, Tawam Hospital, Al Ain, ARE; 3 Medicine, Emirates Medical Association, Dubai, ARE

**Keywords:** hbas-hereditary persistence of fetal hemoglobin, sickle cell trait-hereditary persistence of fetal hemoglobin, hbas-hpfh, hbs-hpfh, sickle cell trait-associated bone disease, bone infarction, sickle cell trait-associated lung disease, hereditary persistence of fetal hemoglobin, medullary bone infarction, sickle cell trait

## Abstract

Sickle cell disease (SCD) is a group of inherited blood disorders characterized by abnormal hemoglobin production, affecting individuals worldwide with varying prevalence across different populations. Manifestations vary, ranging from severe to mild. SCD is characterized by the presence of hemoglobin S (HbS), which distorts erythrocytes upon deoxygenation, leading to sickling. This results in hemolytic anemia, painful vaso-occlusive crises (VOC), and multiple organ damage, including bones, due to microinfarcts. Sickle cell trait (SCT), or carrier status, is not considered an SCD and often runs a benign course.

We report a 44-year-old man of African descent presenting with a one-month history of pain in his ankles and feet. He had a prior diagnosis of sickle cell "trait" without previous VOC. Hematological indices were normal. Hemoglobin electrophoresis showed absent HbA, elevated HbS, elevated HbF, and normal HbA2. X-rays and MRI revealed bilateral bone infarction in diaphyses of right proximal and bilateral distal tibias. Molecular analysis of $&beta;$-globin revealed compound heterozygous hemoglobin S and type 2 deletion of persistence of fetal hemoglobin (HPFH). Pulmonary function tests revealed restrictive lung disease. A literature review from 1946 to May 2024 via PubMed, EMBASE, and Medline was performed, revealing two cases of HbS-HPFH with avascular necrosis affecting the femoral neck were briefly reported more than 60 years ago. Although pulmonary function tests in SCD typically show a mild restrictive pattern with decreased diffusion capacity and rarely an obstructive pattern, no cases of HbS-HPFH were identified. In conclusion, multiple bone infarctions are extremely rare in HbS-HPFH. Lung and bone diseases might be unrecognized in this unique disorder.

## Introduction

Sickle cell disease (SCD) encompasses a heterogeneous group of autosomal recessive disorders. All forms share the same mutation involving the substitution of valine for glutamic acid in the β-globin gene, leading to the production of abnormal sickle hemoglobin (HbS). This mutation causes erythrocytes to distort during deoxygenation (as in high altitude and intense physical exertion), resulting in the formation of rigid, sickled, or crescent-shaped erythrocytes (sickling phenomenon). SCD genotypes include the homozygous mutation HbSS (sickle cell anemia, most common and severe type), and the compound heterozygous mutations such as HbSC (HbS and HbC), HbS-$&beta;$ thalassemia, HbSO Arabia, Hb-SE, HbSD Punjab / Los Angeles, and HbS-$&alpha;$ thalassemia [1]. Sickle cell trait (SCT), or carrier status, is not considered a form of SCD. SCT results from inheriting one sickle cell gene (HbS) from one parent and one normal hemoglobin gene (HbA) from the other, producing hemoglobin AS (HbAS). It often runs a benign course [1]. Similar to SCD, it can co-exist with other hemoglobinopathies, such as $\alpha$-thalassemia [2].

Clinical phenotypes of SCD vary, ranging from hemolytic anemia and painful vaso-occlusive crises (VOC) to multiple organ damage, including bones, due to microinfarcts. The most common complication of SCD is that affecting the musculoskeletal system, primarily bones. Among all the SCD genotypes, bone infarction is most common in patients with sickle cell anemia (HbSS) [1]. The lungs can be affected more by restrictive lung disease than obstructive lung disease, often leading to decreased diffusion capacity [3].

Elevated hemoglobin F (HbF), resulting from deletions or point mutations within the β-globin gene (HBB) cluster and caused by hereditary persistence of fetal Hb (HPFH). These mutations affect the regulation of hemoglobin production, leading to continued production of HbF, contributing to this amelioration. Generally, HbS-HPFH is considered a benign condition [1].

To our knowledge, this is the first detailed case highlighting multiple medullary bone infarctions and asymptomatic restrictive and obstructive lung disease in a patient with persistent elevated HbF and compound heterozygous HbS-HPFH type 2 (Ghana) deletion, as evidenced by genetic analysis.

## Case presentation

A 44-year-old man of African descent was referred to our rheumatology clinic for assessment of bilateral swelling of the ankles. He had a prior diagnosis of sickle cell trait, which had been diagnosed incidentally at the age of 15. He had no history of any episode of VOC. He underwent appendectomy in 1988 with no peri-operative complications. He had two episodes of transient chest pain for which he presented to the emergency department (ED) in 1990 and 2008 with negative cardiac workup, including cardiac enzymes, electrocardiogram (ECG), chest X-rays, and computed tomography angiogram. There was no history of Gaucher disease, Caisson disease (decompression sickness), corticosteroid intake, hyperlipidemia, connective tissue disease, or prior radiation exposure. An HIV test was negative. 

His father and mother died of natural causes at the ages of 110 and 93, respectively. He has six brothers, two of whom have sickle cell "trait", and a half-sister has sickle cell disease (HbSS) but, to his knowledge, without painful VOC. He had had an active life, especially with sports. He was married with healthy children. He was a lifelong non-smoker and did not drink alcohol.

Eight months prior to coming to our rheumatology clinic, he presented to the ED with a four-day history of non-exertional left-sided chest pain and a one-month history of pain in his heels and ankles associated with mild swelling of the ankles, pruritus, and a burning sensation on the dorsum of his feet. There was no antecedent trauma or infection. The pain had worsened two weeks earlier and did not improve on paracetamol (acetaminophen) and codeine. He had no constitutional symptoms. His vital signs were normal. His cardiac workup was negative except for borderline left ventricular hypertrophy on ECG. Table 1 shows laboratory blood tests at different clinic visits. His sickle cell screen was positive.

**Table 1:** Blood tests of our patient at different clinic visits MCV - mean corpuscular volume; MCH - mean corpuscular hemoglobin; MCHC - mean corpuscular hemoglobin concentration

	Normal value	10 years prior to referral	8 months prior to referral	Follow-up after 1 year
Hb, g/dL	14.0 – 18.0		15.7	16.4
Hematocrit, %	42 – 54.0		43.0	45.5
MCV, fL	80.0 – 95.0		81.6	81.3
MCH, pg	27.0 – 32.0		29.5	29.4
MCHC, g/dL	31.0 – 36.0		36.1	36.1
White cell count, cells/mm^3^	4.0 – 11.0		6.3	4.3
Platelets, cells/mm^3^	150 – 400		167	201
Blood film			Normal	Normal
Sickle cell screen	Negative	Positive	Positive	Positive
Hb electrophoresis				
HbA, %	96.5 – 99.9	0	0	0
HbA2, %	2.0 – 3.5	2.9	2	2.5
HbS, %	0	63.3	56	58.1
HbF, %	≤ 3.0	33.8	42	39.4
Reticulocyte count, cells/mm^3^	30 – 110		84	67
Ferritin, µg/L	22 – 275		62	105
Alanine aminotransferase, IU/L	7 – 40		12	11
Aspartate aminotransferase, IU/L	5 – 34		25	19
Alkaline phosphatase, IU/L	40 – 150		75	97
Total bilirubin, mg/dL	≤ 1.3		1.2	1.3
Indirect bilirubin, mg/dL	0 – 1.0			0.9
Albumin, g/dL	3.8 – 5.0			4.3
Lactate dehydrogenase, IU/L	100 – 250		242	234
International normalized ratio	0.80 – 1.24		1.14	0.96
Activated partial thromboplastin time, seconds	26 – 38		30.5	30
C-reactive protein, mg/L	≤ 11.0			< 3
Erythrocyte sedimentation rate, mm/hour	0 - 11		1	0
Creatinine kinase, IU/L	≤ 240		175	148
Creatinine, mg/dL	0.72 – 1.44		1.32	1.11
Uric acid, mg/dL	≤ 7.55		5.51	4.84
Total calcium, mg/dL	8.8 – 10.5			9.4
Phosphate, mg/dL	2.48 – 4.33			2.72
25-hydroxy-vitamin D, nmol/L	61 – 200: optimal levels 25 – 60: mild to moderate deficiency < 25: severe deficiency		61	49
Tissue transglutaminase IgA, CU	< 20			< 2
Homocysteine, µmol/L	5.0 – 15.0			11.8
Antiphospholipid antibodies (anti-cardiolipin IgG and IgM, beta 2 glycoprotein IgG and IgM, and lupus anticoagulant)	Negative			Negative
Antinuclear antibody screen	Negative			Negative

His sickle cell screen was positive. Hemoglobin electrophoresis revealed elevated HbS (56%), elevated HbF (42%), and normal HbA2 (2%). Hemoglobin electrophoresis performed 10 years previously had revealed similar values. There was no evidence of hemolysis (normal complete blood count (CBC), normal blood film, normal aminotransferases, negative serum-free hemoglobin, hemopexin-heme complex, and methemalbumin). He had normal ferritin, 25-hydroxy-vitamin D level (low normal), calcium, fasting blood glucose, creatine kinase, international normalized ratio (INR), and urinalysis. Blood cultures were negative. An antiphospholipid panel was negative. Blood protein S and C levels were normal. Tests for factor V Leiden mutation and MTHFR gene mutation were not conducted. He was admitted to General Medicine with a provisional diagnosis of acute sickle cell crisis. He was initially managed with hydration and morphine, and one dose hydroxyurea. He was discharged home after two days and was referred to a sickle cell clinic for further assessment. At the clinic, he complained of persistent pain in his ankles. The clinical impression was that his symptoms were unrelated to his sickle cell disease, and a referral to a rheumatology clinic was initiated.

When he was seen in the rheumatology clinic, his pain was minimal and he had resumed his regular exercises. There was no history of shortness of breath, transient ischemic attack, stroke, retinopathy, dactylitis, gallstones, frequent urinary tract infections, hematuria, or priapism. He did not have chronic gastrointestinal or respiratory symptoms.

On physical examination, his blood pressure was 110/90, and his heart rate was 68 bpm. His height was 177 cm, his weight was 85.2 kg, and his body mass index (BMI) was 27.2 Kg/m^2^. There was no jaundice or pallor. Examination of the heart, chest, abdomen, and skin was unremarkable. Musculoskeletal examination showed tenderness over joint lines of both ankles and mid-tarsal areas but no evidence of synovitis, tenosynovitis, dactylitis, or enthesitis. A slit-lamp examination by an ophthalmologist did not reveal retinopathy related to sickle cell anemia.

As part of investigating potential lung complications in such patients, pulmonary function tests (PFTs) were ordered, revealing the following spirometry percentages of predicted values: forced expiratory volume in one second (FEV1) at 61%, forced vital capacity (FVC) at 60%, and FEV1/FVC at 80%. The tests also showed low lung volumes, with total lung capacity (TLC) at 67% and residual volume/TLC at 35%. Airway resistance and diffusion capacity (DLCO) were normal. These findings indicate a mixed pattern of restrictive (predominantly) and obstructive lung disease.

**Figure 1:**
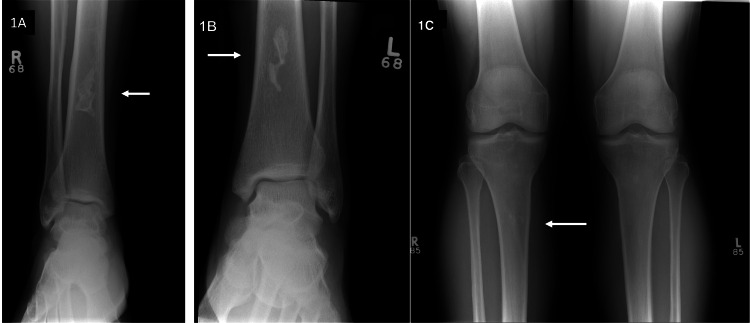
A,B) X-rays of ankles showing bilateral sclerotic lesions (white arrows) within the medullary cavity of distal tibial diaphyses, which are suggestive of intra-medullary infarcts. C) X-ray of the knees revealing a faint geographic sclerotic density within the proximal right tibial meta-diaphysis (white arrow) suspicious for small infarcts

A two-dimensional trans-thoracic echocardiogram was normal, with no evidence of pulmonary hypertension. Chest X-ray was normal. X-rays of the ribs and spine did not show infarction or fractures. X-rays of ankles revealed bilateral sclerotic lesions (white arrows) within the medullary cavity of distal tibial diaphyses, which are suggestive of intra-medullary infarcts (Figures 1A, 1B). X-rays of the knees (Figure 1C) showed a faint geographic sclerotic density within the right proximal tibial meta-diaphysis, suspicious for small infarcts.

**Figure 2:**
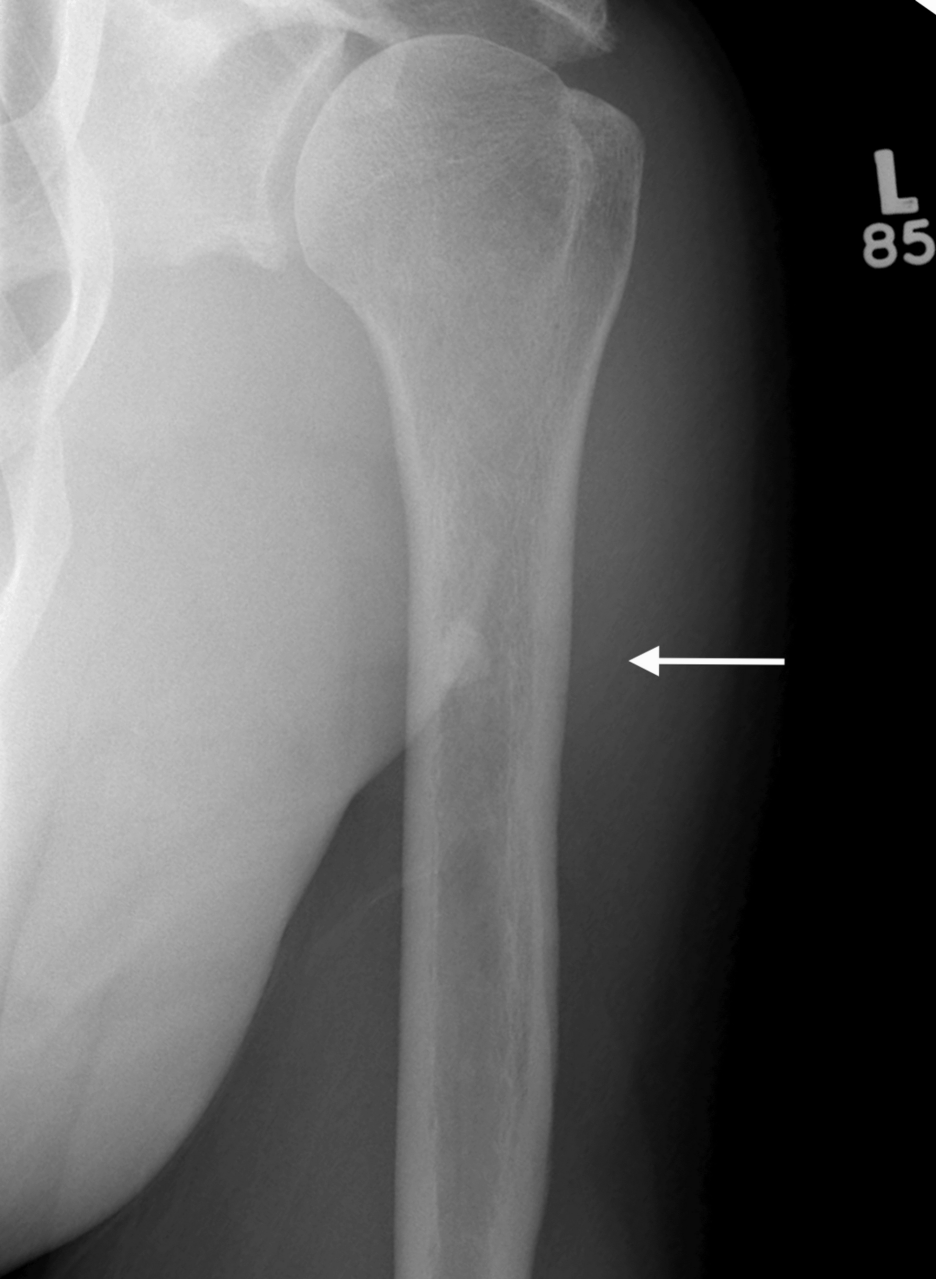
Left shoulder X-ray showing a small medullary infarct (white arrow) at the medial aspect of the proximal humeral shaft

A left shoulder X-ray (Figure 2) revealed a small medullary infarct at the medial aspect of the proximal humeral shaft.

**Figure 3:**
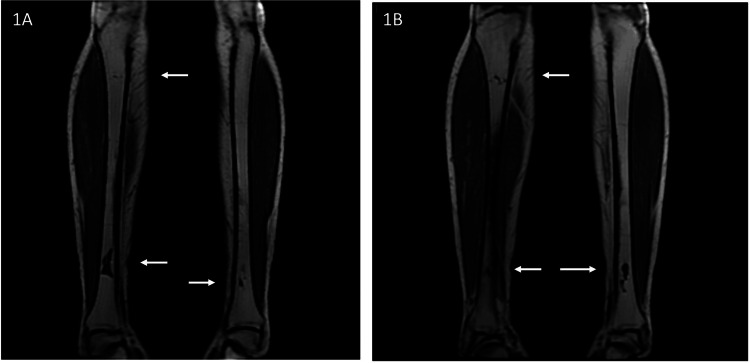
Two consecutive coronal MRI images of the legs on T1-weighted image revealing serpiginous areas (white arrows) of low T1 signal intensity in the medullary cavity of proximal and distal tibial diaphyses bilaterally, suggestive of medullary infarcts

MRI of the legs showed serpiginous areas of low T1 signal intensity and high T2 signal intensity in the medullary cavity of right proximal and bilateral distal tibial diaphyses, suggestive of medullary infarcts (Figure 3).

Dual-energy X-ray absorptiometry (DXA) using a Hologic machine revealed bone mineral density (BMD) of 1.169 g/cm^2^ with a T-score of 0.0 for the lumbar spine (L1-4) and BMD of 1.018 g/cm^2^ with a T-score of - 0.4 for the left hip BMD. The 10-year probability of a fracture was 1.9%, while the probability of a hip fracture was 0%.

Molecular tests of hemoglobin included $\beta$-globin gene cluster analysis by polymerase chain reaction-amplification refractory mutation system (PCR-ARMS) and deletion-specific PCR. These tests confirmed a compound heterozygous state for the HbS mutation (GAG>GTG) in codon 6 of the β-globin gene (HBB c.20A>T) and the HPFH type 2 (Ghanaian) deletion.

Since his symptoms were mild, he was prescribed meloxicam 7.5 mg orally twice daily, and calcium and vitamin D 1000 units orally daily. He initially used meloxicam as needed, as his ankle symptoms completely resolved shortly.

The patient followed up at the rheumatology clinic after one year. He was still asymptomatic. A repeat CBC and Hb electrophoresis were unchanged. Hemolytic markers were still negative. His fasting lipids and coagulation profile were normal. 

## Discussion

Sickle cell trait (SCT) or HbAS is present in approximately 8% of African Americans [1]. The two major aspects that highlight this case are bone health and lung disease.

Bone infarction and avascular necrosis in HbS-HPFH

The underlying cause of SCD seems to be related to VOC, which can result in bone infarction or avascular necrosis (AVN), osteomyelitis, fractures, osteoporosis, and dental changes. AVN generally denotes ischemia and necrosis of the epiphyses of the heads of long bones, including the femoral and shoulder heads, knees, and small joints of the hand and feet (hand-foot syndrome) [4]. Vertebral collapse can complicate infarction, resulting in a biconcave ("fish mouth") appearance. Orbital collapse and compression can also complicate infarction, causing sudden periorbital swelling.

Bone infarction is a term used to indicate a consequence of ischemia occurring in metaphyses and diaphyses of long bones, most commonly the tibia and fibula. Typical clinical features of bone infarction are severe pain, mild localized erythema and warmth, and sometimes low-grade fever and leukocytosis [4]. In the acute setting of bone infarction, X-rays may appear normal. Therefore, MRI with T1- and T2-weighted images can be used for early diagnosis. On T1-weighted (T1W) MR images, a ring-shaped or serpiginous pattern of low signal intensity may be seen, while on T2-weighted (T2W) images, there may be variable signal intensity, sometimes displaying a double line appearance [5]. In subacute or chronic settings, X-rays may reveal dense calcifications (with marked sclerotic margin) either as focal and geographic (serpiginous) or diffuse and patchy sclerosis [4].

The main differential diagnosis includes a chronic healing non-aggressive process such as non-ossifying fibroma (NOF). NOF, or fibroxanthoma, is a benign bone tumor commonly found in children and adolescents. It typically occurs in the metaphyseal regions of long bones, with the distal femur and proximal tibia being the most frequent sites. NOF is usually asymptomatic and often discovered incidentally on X-rays taken for other reasons [6]. If symptomatic, the most common symptoms are pain and swelling at the affected bone. NOF can rarely occur at multiple sites [7]. On X-rays, NOF appears as a dark mass with a thin surrounding white border. It is not associated with intramedullary bone infarction.

Malignant transformation of bone infarcts occurs very rarely (estimated to be 1% of all bone sarcomas) and typically presents as a solitary lesion affecting long bones. Although case reports described this in SCD, including sickle cell anemia (HbSS) or trait (HbAS), it has not been reported in HbS-HPFH. Such malignancies include malignant fibrous histiocytoma (also known as undifferentiated pleomorphic sarcoma), osteosarcoma, and fibrosarcoma [8-10]. Our patient had atypical symptoms with mild pain and swelling at the ankles and a burning sensation on the dorsum of the feet. There is no clinical or radiological evidence of tumors.

The severity of SCD is affected by the presence of some favorable prognostic factors such as HBB chromosomal haplotypes, such as the four African haplotypes, elevated HbF level, single nucleotide polymorphism (SNPs) genetic modifiers, especially with Xmn1 or Hpal, and co-inherited thalassemias such as $&alpha;$-thalassemia [11,12]. The Hb electrophoresis of our patient is unique in that HbS and HbF levels were both elevated and yet he had multiple bone infarctions.

Elevated HbF ($\alpha$2$\gamma$2) appears to ameliorate or even prevent VOC via a number of mechanisms. First, HbF is highly concentrated in erythrocytes, hence it dilutes HbS, consequently inhibiting HbS ($\alpha$2$\beta$2S) polymerization. Secondly, erythrocytes with HbF, referred to as F cells, live longer than non-F cells, likely reflecting their resistance to mechanical trauma. Thirdly, F cells do not participate in the irreversible sickling process as opposed to the sickled cells, which are prematurely captured and destroyed in the spleen [13,14]. Further, high HbF was found to predict longer survival in patients with SCD [15]. Recently, the presence of F cells in SCD was found to be associated with decreased thrombin formation [16]. Therefore, increasing HbF levels is the goal of using hydroxyurea in the prevention of VOC. One of the main causes of elevated HbF is HPFH, which is a heterogeneous group of inherited diseases that was discovered in Ghana in 1955 in patients with SCD [17] and is characterized by elevation of HbF beyond infant life. HPFH genotypes include heterozygotes, homozygotes, and compound heterozygotes with other hemoglobinopathies such as SCD, SCT, or thalassemias. The true prevalence of compound heterozygotes of HbS-HPFH is unknown due to a lack of population-based studies. The incidence of HPFH heterozygotes was 0.2% in African Americans in series from Johns Hopkins, although molecular testing was not performed [18]. With advances in molecular genetics, the cause was identified as mutations that prevent switching HbF to the adult HbA ($\alpha$2$\beta$2), which normally occurs by six months of life. These mutations can be either due to deletion of the HBB cluster or non-deletion (point mutations) of the $\gamma$-globin gene (HBG) promotor regions or quantitative trait loci. The deletional type 1 (African and African American) and 2 (Ghanaian) mutations result in the pancellular distribution of HbF among all RBCs, compared to the non-deletional type, which is heterocellularly distributed [19]. The mean HbF level of the heterozygous deletional types 1 and 2 is 20-28%, and hematological parameters (e.g., mean corpuscular volume) are normal [14,20]. In our patient, HbF levels ranged between 33.8 to 42%.

The benign clinical course of patients with the compound heterozygous HbS-HPFH genotype has been emphasized in the literature. Conley et al. [21] found that such patients were generally in good health without symptomatic VOC, anemia, or hemolysis. HbF was, as the deletional type of HPFH, uniformly distributed among all RBCs and ranged between 15-35%. A more recent study of 30 HbS-HPFH patients who were identified as having HbF levels of >10% in patients with sickle cell anemia. Patients had a mean age of 6.1 ± 6 years with near-normal Hb levels of 12.7 ± 11 g/dL, mild microcytosis (average mean MCV 74.6 ± 6.3 fL), and an average HbF of 31.2 ± 2.4% for those ≥5 years of age. These patients also did not have symptoms of VOC. In addition, HbF was at its highest level at birth (50-90%) and then started to decline before stabilizing at three to five years of age (30%). However, HbF level was monitored and detectable until the age of 20. This was the first largest study describing the clinical and laboratory features with molecular analysis of this disease [20].

We did not test for the MTHFR gene in our patient, as it has been shown to have little association with osteonecrosis in patients with SCA [22]. The exact reason for our patient's multiple bone infarctions and mixed restrictive and obstructive lung disease, despite having a high HbF level and the African haplotype, remains unclear. However, several factors could influence HbF's efficacy in inhibiting HbS polymerization. These include HbF distribution (pancellular or heterocellular), best measured by flow cytometry, the total amount of HbF per cell, and aging [23,24]. Interestingly, some ethnicities and specific haplotypes (e.g., Arabian-Indian or Benin haplotype) may play a role in the development of end-organ damage, such as osteonecrosis and lung disease. This has been observed in studies involving Saudi and Kuwaiti patients with high HbF and SCA, either alone or in combination with other hemoglobinopathies [25-27]. Furthermore, the Cooperative Study of Sickle Cell Disease (CSSCD) DNA samples have identified certain candidate gene SNPs (e.g., BMP6, annexin A2, and klotho) as risk factors for the development of avascular necrosis of the hip and/or shoulder [28]. Although the above studies did not specifically address compound heterozygous HbS-HPFH, these insights suggest that our patient's unique genetic background and HbF characteristics could contribute to the severity of his condition, underscoring the need for further research in this area.

A literature review from 1946 to May 2024 via PubMed, EMBASE, and Medline was performed using various combinations of the following keywords: "sickle cell disease", "hereditary persistence of fetal hemoglobin", "bone infarction", "osteonecrosis", "lung disease", and "bone disease". Relevant articles were retrieved based on the title and/or abstract if available. If an article cited other relevant article(s), the latter was reviewed to identify potential items. 

Two cases of possible association between SCT with HPFH (HbS-HPFH) and AVN of femoral heads were identified. The first case, reported in 1958, was an 18-year-old man from Uganda who complained of an eight-year history of bilateral hip pain, which had been attributed to Perthe's disease until radiological findings suggested SCD. However, bone infarction and AVN were not mentioned. His HbF level was elevated at 15%, while his mother's was 22%. His father and sister had sickle cell trait (HbAS) [29]. The second case, published in 1963, reported briefly that a patient had compound heterozygous HbS-HPFH and AVN of the femoral head [21].

Evidence is lacking on the management of bone infarction in HbS-HPFH, but non-steroidal anti-inflammatory drugs can be tried to treat symptoms such as pain [30].

Bone density in sickle cell trait (SCT)

Despite a lack of evidence of an association between compound heterozygous HbS-HPFH and bone disease, approximately 80% of adults with SCD are found to have abnormal BMD. These abnormalities are associated with low BMI, low Hb level, and high ferritin, which our patient did not have [31]. 

Lung diseases in SCD and SCT

PFTs often show a mild restrictive pattern with decreased diffusion capacity, although an obstructive pattern has been observed in some patients with SCD. The cross-sectional multi-center CSSCD study found abnormal PFTs in 90% of African American patients with sickle cell anemia (HbSS), with only five out of 270 patients demonstrating mixed restrictive and obstructive lung disease [3]. A recent study followed PFTs of 92 adults with SCD for a mean of 13 years and showed a decline of FEV1 that was independent of cigarette smoking or the frequency of VOC. Among the SCD phenotypes, there were only two patients with HbS-HPFH whose PFT pattern was not specified. Patients with HbSS and those with HbS-HPFT experienced a comparable decline in FEV1 [32]. Our patient did not complain of chronic respiratory symptoms, was a lifetime non-smoker, and had PFT findings that showed restrictive more than obstructive lung disease with normal DLCO, which are not related to occupational exposures. Similarly, evidence of the association between SCT or HbS-HPFH and pulmonary hypertension is lacking.

## Conclusions

We present the first detailed patient case demonstrating multiple bone infarctions and restrictive more than obstructive lung disease in a young man with compound heterozygous HbS-HPFH. Despite the general belief that such patients with sickle cell trait typically have a benign course, those who experience pain and/or swelling localized to a long bone or deep-seated joint such as the hip should undergo clinical and radiological evaluation to rule out bone infarction. Additionally, assessment of bone disease and lung abnormalities is also recommended based on the patient's symptoms. Asymptomatic abnormalities of the bone and lung, as seen in our patient, might be unrecognized. By shedding light on this unique genetic combination, this case broadens our understanding of the diverse clinical spectrum of SCD, emphasizing the significant role of elevated HbF in modifying disease severity.
